# SRMDAP: SimRank and Density-Based Clustering Recommender Model for miRNA-Disease Association Prediction

**DOI:** 10.1155/2018/5747489

**Published:** 2018-03-21

**Authors:** Xiaoying Li, Yaping Lin, Changlong Gu, Zejun Li

**Affiliations:** ^1^College of Computer Science and Electronic Engineering, Hunan University, Changsha 410082, China; ^2^Key Laboratory of Trusted Computing and Networks, Hunan Province, Changsha 410082, China; ^3^School of Computer and Information Science, Hunan Institute of Technology, Hengyang 412002, China

## Abstract

Aberrant expression of microRNAs (miRNAs) can be applied for the diagnosis, prognosis, and treatment of human diseases. Identifying the relationship between miRNA and human disease is important to further investigate the pathogenesis of human diseases. However, experimental identification of the associations between diseases and miRNAs is time-consuming and expensive. Computational methods are efficient approaches to determine the potential associations between diseases and miRNAs. This paper presents a new computational method based on the SimRank and density-based clustering recommender model for miRNA-disease associations prediction (SRMDAP). The AUC of 0.8838 based on leave-one-out cross-validation and case studies suggested the excellent performance of the SRMDAP in predicting miRNA-disease associations. SRMDAP could also predict diseases without any related miRNAs and miRNAs without any related diseases.

## 1. Introduction

MicroRNAs (miRNAs) are small endogenous noncoding RNAs which are approximately 22nt long. Since the discovery of the first two miRNAs lin-4 and let-7, thousands of miRNAs have been identified in eukaryotic cells [1, 2]. A series of studies have shown that miRNAs play an important role in many biological processes, such as cell growth and apoptosis, proliferation, differentiation, and signal transduction [3–6]. Given that miRNAs are involved in the normal function of cells, aberrant miRNA expression has been associated with many types of human diseases, ranging from common diseases to cancers [7–9]. Therefore, the identification of disease-related miRNAs is beneficial in understanding the molecular mechanism of the disease pathogenesis and disease diagnosis and to further promote the level of treatment and prevention.

To date, many biological experimentations have been performed to determine a large number of miRNA-disease associations. Many studies have built databases, such as HMDD [10], miR2Disease [11], dbDEMC [12], miRCancer [13], and PhenomiR [14], to serve as a solid data foundation for predicting miRNA-disease associations. HMDD is a database manually retrieved from the literature [10]. The latest version is HMDD v2.0, which integrates 10,368 miRNA-disease associations of approximately 572 miRNA genes and 378 diseases from 3,511 papers. MiR2Disease documents 1,939 manually curated miRNA-disease associations between 299 human miRNAs and 94 human diseases [11]. The dbDEMC stores differentially expressed miRNAs in human cancers obtained from microarray data [12]. The updated version dbDEMC 2.0 contains 2,224 differentially expressed miRNAs in 36 cancer types [15]. The miRCancer stores miRNA-cancer associations obtained by text mining method [13]. PhenomiR provides information about differentially regulated miRNA expression in diseases and other biological processes [14].

However, using experimental methods to identify the disease-related miRNAs is time-consuming and costly. Based on existing data, computational methods have been developed as a valuable supplement to the experimental methods to save experimental time and cost. Computational methods can calculate and rank the similarity scores of all miRNAs for a given disease. Top-ranked miRNAs are treated as the most promising candidate disease miRNAs for further experimental studies. Similarity calculation is the key issue in computational methods [16]. According to the calculation of similarity score, most computational methods are divided into two categories [17, 18], namely, network-based methods [19–28] and machine-learning-based methods [24, 29–34]. Network-based methods predict miRNA-disease associations by considering the hypothesis that miRNAs with similar functions usually tend to be associated with phenotypically similar diseases [10]. Jiang et al. [19] constructed a human phenome-miRNAome functional association miRNA network using the hypergeometric distribution scoring system to select the candidate disease miRNAs. However, high final prediction accuracy may not be obtained if only the local information of each miRNA is issued and the study is strongly dependent on the predicted miRNA-target interactions. Chen et al. [21] adopted global network similarity measures and developed RWRMDA to infer the associations between diseases and miRNAs by implementing random walk on the miRNA-miRNA function similarity network. Based on the weighted k most similar neighbors, Xuan et al. [22] proposed HDMP to infer disease-related miRNAs. HDMP evaluates miRNA function similarity by incorporating the information content of disease terms, disease phenotype similarity, and weight information of the miRNA family or cluster. However, RWRMDA and HDMP cannot be useful for predicting disease without any related miRNAs. Based on social network analysis, Zou et al. [24] proposed KATZ method to compute the similarity score based on walks of different lengths between the miRNA and disease nodes. However, KATZ has relatively poor capability of sparing known associations. Gu et al. [25] calculated miRNA similarity and disease similarity of known miRNA-disease associations through the Jaccard similarity measure. They incorporated miRNA similarity of known miRNA-disease associations, miRNA functional similarity, and miRNA family information to construct miRNA similarity network and incorporated disease similarity of known miRNA-disease associations to construct disease similarity network. Then, they applied network consistency projection method to predict the disease-related miRNAs.

Machine-learning-based methods extract features from data to initially obtain effective features of miRNAs and diseases and then utilize machine learning models to predict miRNA-disease associations. Jiang et al. [29] showed a support vector machine (SVM) classifier method by integrating the feature vectors of miRNA-target and phenotype similarity. Xu et al. [31] introduced an approach based on the miRNA-target-dysregulated network to prioritize novel disease miRNAs. This method also constructs a support vector machine classifier based on the features and changes in miRNA expression. However, these two computational methods are mainly limited by the difficulty or impossibility of obtaining negative training samples, and this drawback would largely influence the predictive accuracy. To solve this problem, Chen and Yan [30] developed a semisupervised method of regularized least squares for miRNA-disease association (RLSMDA). RLSMDA integrates known disease-miRNA associations, disease similarity dataset, and miRNA functional similarity network to infer potential disease-related miRNAs. The main drawback of RLSMDA is the intricate adjustment of parameters. Xiao et al. [35] used graph-regularized nonnegative matrix factorization framework to predict potential miRNA-disease associations using weighted *k* nearest neighbor profiles to incorporate miRNA similarity and disease matrices. Chen et al. [34] presented a computational method DRMDA based on stacked autoencoder, greedy layer-wise unsupervised pretraining algorithm and SVM, and this method was implemented to predict potential miRNA-disease associations. However, DRMDA results are not highly accurate, because of the difficulty in obtaining negative samples and optimizing the complex parameters.

Similarity calculation mainly considers miRNA-miRNA similarity measurement. Several computational methods use the known miRNA-disease associations in calculating miRNA-miRNA similarity [19–26, 29, 30]. In these methods, miRNA-miRNA similarity measurement is completed by disease-disease measurement and known experimental miRNA-disease associations. However, these methods are restricted by the possible overestimation of the predictive accuracy. This drawback may be due to the fact that cross-validation experiments are not correctly performed, and the miRNA-miRNA similarity depends heavily on the known miRNA-disease associations. These methods fail to remove known information of the tested element for similarity calculation at each round of cross-validation. Other limitations include the inability to predict isolated miRNA and lack of disease semantic similarity [36]. An isolated miRNA signifies that a miRNA has no associated disease; that is, no relationship exists between this isolated miRNA and diseases. Thus, miRNA-disease associations cannot be used to calculate miRNA similarity of an isolated miRNA. Instead of using experimentally verified miRNA-disease associations, other computational methods calculate miRNA similarity using the interaction of miRNAs with other biomolecules [31, 36–38]. For example, Liu et al. [36] calculated miRNA similarity using the miRNA-target gene and miRNA-long noncoding RNA associations. However, the performances of these methods are deficient.

Based on the assumption that miRNAs with similar functions are normally associated with phenotypically similar diseases and vice versa, we solved the aforementioned limitations by establishing a novel computational method based on SimRank [39] and density-based clustering [40] recommender model for miRNA-disease association prediction (SRMDAP). The SRMDAP constructs miRNA similarity subnetwork using SimRank to calculate network topological similarity between miRNAs based on miRNA-message RNA (mRNA) interaction network. The disease similarity subnetwork is similar to miRNA similarity subnetwork and is based on the disease-gene network. Then, the SRMDAP uses the density-based clustering recommender model to integrate miRNA similarity subnetwork, disease similarity subnetwork, and experimentally verified miRNA-disease associations to predict potential associations between miRNAs and diseases. In this work, leave-one-out cross-validation experiment and case studies about two important cancers, namely, kidney and colorectal neoplasms, have indicated the excellent predictive performance of SRMDAP. The SRMDAP can also predict isolated diseases and isolated miRNAs.

## 2. Methods

### 2.1. Data

Three datasets were used in our approach. Experimentally verified miRNA-mRNA interactions were downloaded from the miRTarBase database to construct the miRNA similarity network [41] (http://mirtarbase.mbc.nctu.edu.tw/, Release 6.0: Sept-15-2015). Meanwhile, experimentally verified disease-related mRNAs were downloaded from the DisGeNET database [42] (http://www.disgenet.org/web/DisGeNET/menu/home, DisGeNET 4.0: October 2016) to construct a disease similarity network. Experimentally verified miRNA-disease network was downloaded from the HMDD v2.0 database [43] (http://www.cuilab.cn/hmdd, Jun-14-2014 Version).

### 2.2. Data Processing

#### 2.2.1. MiRNA-Disease Association Network

The disease names of the DisGeNET and HMDD databases were mapped to the MeSH description (https://www.ncbi.nlm.nih.gov/mesh). Diseases in the HMDD database not found in the DisGeNET database and repeated associations were removed. Then, we obtained 5,048 known miRNA-disease associations, including 475 miRNAs and 334 diseases, as the benchmark dataset. Formally, we denoted the miRNA set as **M** = {**m**_1_, **m**_2_,…, **m**_**nm**_} and the disease set as **D** = {**d**_1_, **d**_2_,…, **d**_**nd**_}. The variables **nm** and **nd** denote the number of miRNAs and diseases, respectively. Matrix **AS** represents the adjacency matrix of miRNA-disease associations. **AS**(**i**, **j**) = 1 denotes miRNA **i** associated with disease **j**; otherwise, **AS**(**i**, **j**) = 0.

#### 2.2.2. MiRNA Similarity Network

SimRank [39] was employed to calculate the disease and miRNA similarities based on miRNA-mRNA interaction network and disease-related mRNA associations. SimRank is a model to measure the degree of similarity between any two objects on the basis of the information of the topology graph, which has been successfully applied to web page ranking [44], recommender systems [45], outlier detection [46], network graph clustering [47], and approximate query processing [48], among others. The SimRank model defines the similarity of two nodes based on a recursive thinking. When other nodes pointing to the two nodes are similar, then the two nodes are similar. SimRank defines the similarity of two nodes as follows:

(1) sa,b=1a=bCIa·Ib∑j∈Ib ∑i∈Iasi,ja≠b0Ia=∅  or  Ib=∅,

where **s**(**a**, **b**) is the similarity between nodes **a** and **b** and **C** ∈ [0, 1] is a decay factor. **I**(**a**) denotes all node sets that point to node **a**, and |**I**(**a**)| is the number of elements of **I**(**a**).

The adjacency matrix of the miRNA-mRNA interaction bipartite network is represented as **A**, where **A**(**i**, **j**) in row **i** and column **j** is 1 if miRNA **i** is associated with mRNA **j**, and 0 otherwise. The matrix **A** is normalized by column to determine the matrix **W**_1_, and the similarity matrix can be calculated as follows:

(2) $$\begin{array}{c} SM=C_{1}\cdot \left(\;{W_{1}}^{T}\cdot SM\cdot W_{1}\;\right)+\left(\;1-C_{1}\;\right)\cdot I, \end{array}$$

where **SM** is the miRNA similarity matrix and **SM**(**i**, **j**) is the similarity between miRNAs **i** and **j**. **W**_1_^**T**^ is the transpose matrix of **W**_1_, **C**_1_ is a decay factor, and **I** is the unit matrix.

#### 2.2.3. Disease Similarity Network

**Figure 1:**
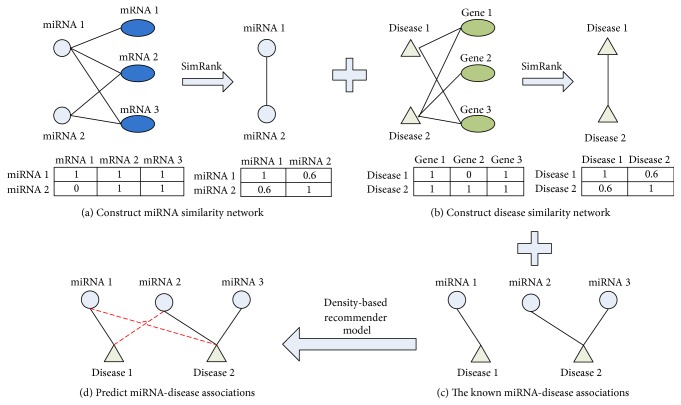
Illustration of the process of constructing miRNA and disease similarity network and predicting miRNA-disease associations. (a) A simple example of constructing similarity of miRNAs 1 and 2 is shown in (a). (b) A simple example of constructing similarity of diseases 1 and 2 is shown in (b). (c) The known miRNA-disease associations. (d) Predicting miRNA-disease associations through density-based recommender model by integrating miRNA similarity network, disease similarity network, and the known miRNA-disease associations.

We can obtain the similarity matrix of diseases using the same process in determining the miRNA similarity network. The adjacency matrix of the disease-gene network is represented as **B**, where **B**(**i**, **j**) in row **i** and column **j** is 1 if the disease **i** is associated with gene **j**, and 0 otherwise. Matrix **B** is normalized by column to obtain the matrix **W**_2_, and the similarity matrix can be calculated as follows:

(3) $$\begin{array}{c} SD=C_{2}\cdot \left(\;{W_{2}}^{T}\cdot SD\cdot W_{2}\;\right)+\left(\;1-C_{2}\;\right)\cdot I, \end{array}$$

where **SD** is the disease similarity matrix and **SD**(**i**, **j**) is the similarity between diseases **i** and **j**. **W**_2_^**T**^ is the transpose matrix of **W**_2_, **C**_2_ is a decay factor, and **I** is the unit matrix. A simple example of constructing miRNA and disease similarity is provided in Figure 1.

### 2.3. Prediction Method

**Figure 2:**
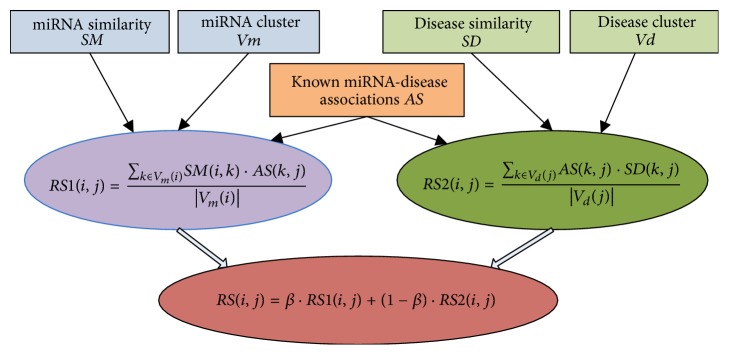
The flowchart of SRMDAP.

In this work, a density-based clustering recommendation model is developed based on the miRNA and disease similarity network to predict potential miRNA-disease associations. The flowchart of SRMDAP is shown in Figure 2.

For example, the calculation for predicting the association of miRNA *i* and disease *j* is as follows. First, given the assumption that miRNAs with similar functions are normally associated with phenotypically similar diseases and vice versa [10, 49], the closer the neighbors of miRNA *i* are to disease *j*, the closer miRNA *i* will be to disease *j* in the miRNA similarity network. Using miRNA *i* as cluster center and greedy method, we added the most similar neighbor nodes to form new clusters, until the cluster density no longer increased. The cluster density of cluster *V* is defined as follows:

(4) $$\begin{array}{c} d\left(\;V\;\right)=\frac{w^{in}}{w^{in}+w^{out}+\alpha \cdot \left|V\right|}, \end{array}$$

where *w*^in^ and *w*^out^ denote the sum of the weights of inner and external sides of cluster *V*, respectively [50]. Item *α* · |*V*| is a penalty item, and |*V*| is the number of members of cluster *V*. In our experiments, we set *α* = 2. Then, using *V*_*m*_(*i*) = [*m*_1_, *m*_2_,…, *m*_*n*_], which denotes the closest neighbors of miRNA *i*, the predictive score between miRNA *i* and disease *j* is calculated as follows:

(5) $$\begin{array}{c} RS1\left(\;i,j\;\right)=\frac{\sum_{k\in V_{m}\left(i\right)}SM\left(i,k\right)\cdot AS\left(k,j\right)}{\left|V_{m}\left(i\right)\right|}, \end{array}$$

where *RS*1(*i*, *j*) is the predictive score between miRNA *i* and disease *j* calculated by the neighbors of miRNA *i*; and *SM*(*i*, *k*) is the similarity of miRNA *i* and miRNA *k*; and *AS*(*k*, *j*) is the association between miRNA *k* and disease *j*. Equation (5) calculates the predictive score based on the nearest neighbors of miRNA *i* and the associations between the neighbors and disease *j*.

Second, in the same way, based on the assumption that diseases with similar functions often have similar semantic descriptions and vice versa [20], the closer the neighbors of disease *j* are to miRNA *i*, the closer the disease *j* will be to miRNA *i* in the disease similarity network; the predictive score between miRNA *i* and disease *j* is calculated as follows:

(6) $$\begin{array}{c} RS2\left(\;i,j\;\right)=\frac{\sum_{k\in V_{d}\left(j\right)}AS\left(k,j\right)\cdot SD\left(k,j\right)}{\left|V_{d}\left(j\right)\right|}, \end{array}$$

where *V*_*d*_(*j*) is the closest neighbor to disease *j*.

Finally, the final predictive score between miRNA *i* and disease *j* is calculated by integrating *RS*1(*i*, *j*) and *RS*2(*i*, *j*) as follows:

(7) $$\begin{array}{c} RS\left(\;i,j\;\right)=\beta \cdot RS1\left(\;i,j\;\right)+\left(\;1-\beta \;\right)\cdot RS2\left(\;i,j\;\right), \end{array}$$

where *β* ∈ [0,1] is an integration parameter to balance the contributions from miRNA and disease similarities. *RS*(*i*, *j*) in row *i* and column *j* is the prediction value of miRNA *i* to disease *j*.

When the predictive score between isolated disease *j* and miRNA *i* is calculated, all associations of isolated disease *j* are ignored, and the contribution of the neighbors of miRNA *i* to the predictor is zero. Thus, *RS*1(*i*, *j*) equals 0. The final predictive score between isolated disease *j* and miRNA *i* is *RS*2(*i*, *j*), which is the predictive score between the similarity neighbors of disease *j* and miRNA *i*. Therefore, SRMDAP can predict associated miRNAs for an isolated disease. Similarly, when the predictive score between new miRNA and disease is calculated, *RS*1(*i*, *j*) is the predictive score between the similarity neighbors of miRNA *i* and disease *j*, and only *RS*1(*i*, *j*) is used as the predictive score between the new miRNA and related diseases.

**Figure 3:**
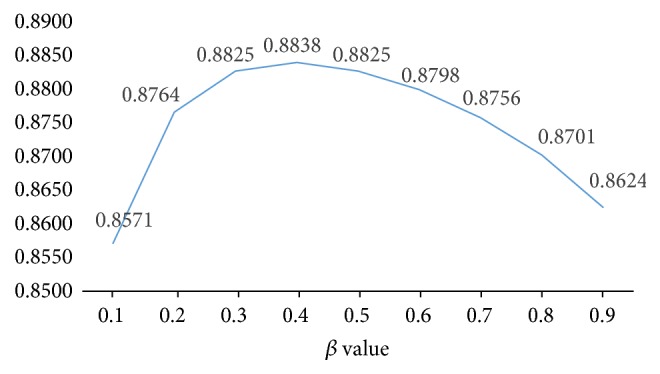
Average AUCs affected by *β* value. When *β* is 0.4, average AUC is 0.8838 and SRMDAP achieves the best performance.

To explore for a suitable *β* value, we tested different *β* values from 0.1 to 0.9 and calculated the average area under the curve (AUC) in the framework of leave-one-out cross-validation. The results showed that SRMDAP achieved the highest average AUCs when *β* was 0.4 (Figure 3).

## 3. Results

### 3.1. Characteristics of the miRNA-Disease Association Network

**Figure 4:**
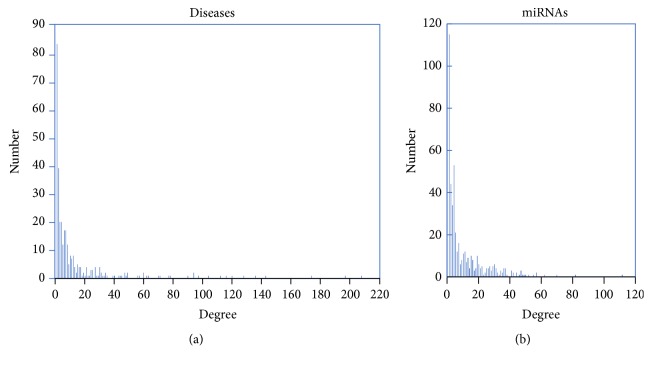
Disease degree distribution and miRNAs degree distribution in the known miRNA-disease association network. (a) shows the bar diagram of disease degree. (b) shows the bar diagram of miRNAs degree.

In our study, 5,048 known miRNA-disease associations consisting of 475 miRNAs and 334 diseases were included. To comprehensively illustrate the known miRNA-disease association network, we demonstrated the characteristics of known miRNA-disease association network in Table 1. The degree of a disease (or miRNA) represented the neighboring miRNAs (or disease) related to it. The average degrees of the disease and miRNAs were 15.11 and 10.63, respectively. The degree of distribution of diseases and miRNAs of the known miRNA-disease association network (Figure 4) revealed a power-law distribution. Most of the miRNAs and diseases presented a degree of 1. Hepatocellular carcinoma showed that the maximum degree, that is, 208 miRNAs, was related to this malignancy. Meanwhile hsa-mir-21 showed the maximum degree, with 112 diseases related to this miRNA.

### 3.2. Performance Evaluation of SRMDAP

We implemented the leave-one-out cross-validation (LOOCV) on the known miRNA-disease associations to evaluate the predictive performance of the SRMDAP. For a given disease *d*, each known association between miRNA and disease *d* was ignored in turn as a test sample, and other known associations between miRNAs and disease *d* were considered as a training set. The remaining miRNAs without evidence to show their relation to disease *d* composed the candidate miRNA set. We calculated the relevance score of these candidate miRNAs with disease *d* and ranked them by their scores. If the rank exceeded a given threshold, then the SRMDAP model successfully predicted this miRNA-disease association. The threshold was varied to draw the receiver operating characteristic (ROC) curve, and the score of the AUC was calculated to demonstrate the predictive performance. The ROC plots the relationship between the true positive rate (TPR, sensitivity) and the false positive rate (FPR, 1 − specificity) at different thresholds. Sensitivity represents the percentage of test miRNA-disease associations with ranking above a given threshold. Meanwhile, specificity represents the percentage of miRNA-disease associations below the threshold.

The TPR and FPR were calculated as follows:

(8) TPR=TPTP+FN,FPR=FPTN+FP,

where TP, FP, TN, and FN indicate true positive, false positive, true negative, and false negative, respectively. Given a threshold, TP and FP are the number of known and unknown associations above the threshold, respectively. TN and FP are the number of unknown and known associations below the threshold, respectively. The AUC value of 1 indicates perfect performance of the prediction method. Moreover, an AUC value of 0.5 implies the random performance of the prediction method.

**Figure 5:**
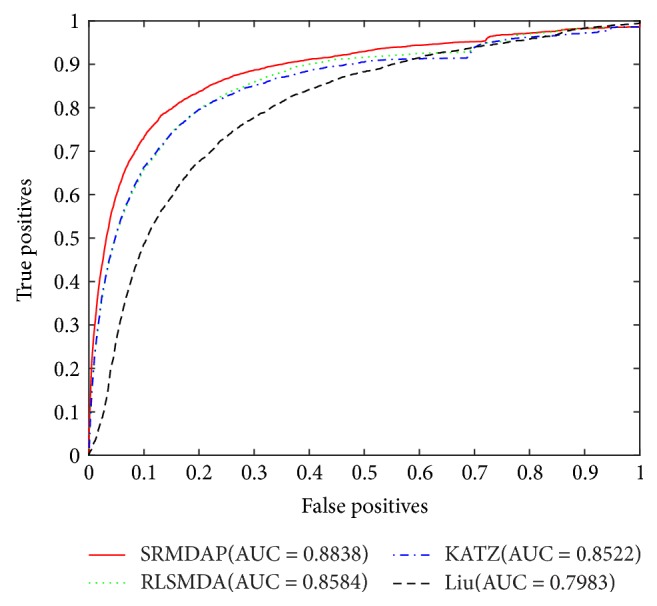
Method comparison: comparison between SRMDAP, RLSMDA, KATZ, and Liu's method in terms of ROC curve and AUC.

To our knowledge, RLSMDA [30], KATZ [24], and Liu et al.'s method [36] are three the-state-of-the-art computation methods that predict miRNA-disease associations. In our work, we compared SRMDAP with these methods and implemented a LOOCV for the three methods. The SRMDAP achieved the highest AUC of 0.8838 when *β* = 0.4. When optimal parameters were selected as described by the authors, AUC values corresponding to RLSMDA, KATZ, and Liu's method were 0.8584, 0.8522, and 0.7983, respectively. Comparative results of overall ROC curves and AUCs of all methods are shown in Figure 5.

To obtain a reliable judgment, we tested 18 human diseases associated with at least 70 miRNAs, because diseases related to a few miRNAs were not sufficient to evaluate the performance of the prediction methods.  Table 2 shows that the SRMDAP achieved the highest AUC of 0.8874 with lung neoplasms and lowest AUC of 0.7367 with renal cell carcinoma. The average AUC value for the 18 diseases was 0.8056. The average AUC values for the 18 diseases obtained from RLAMDA, KATA, and Liu's method were 0.6671, 0.6901, and 0.5178, respectively. The average AUC achieved by SRMDAP was 14%, 12%, and 29% higher than those of the other three methods, respectively. The AUC values of the SRMDAP for the 18 diseases were all higher than those of RLSMDA, KATZ, and Liu's method. These facts indicated that the prediction performance of SRMDAP was superior to RLSMDA, KATZ, and Liu's method.

### 3.3. Case Studies

To further evaluate the SRMDAP's ability to discover potential miRNA-disease associations, we selected two important diseases (kidney neoplasms and colorectal neoplasms) as case studies. We analyzed the top 50 candidates in detail. Prediction results were supported by dbDEMC [15] database and literature.

Kidney neoplasm, which forms in tissues of the kidneys, is one of the top 10 cancer killers. This malignancy is still difficult to diagnose and treat. Based on 2010–2014 cases and deaths, the annual number of new cases of kidney and renal pelvis cancer was 15.6 per 100,000 persons. The five-year survival rate in the United State is 74.1% [51]. MiRNAs showing altered expression in the kidney are promising biomarkers for diagnosis. For example, miR-141 and miR-200b are underexpressed in renal cell carcinoma (a kidney neoplasm type) from normal kidney and oncocytoma in tissue samples. The miRNA expression profiles of miR-141 or miR-200b might provide an ancillary tool for the correct discrimination of kidney neoplasms [52]. Candidate miRNAs were ranked based on the SRMDAP. The top 50 potential miRNAs associated with kidney neoplasms and evidence for the associations with kidney are listed in Table 3. Among the top 50 predicted candidates, 49 miRNA have been confirmed by dbDEMC, and only hsa-mir-7 is not confirmed by dbDEMC. However, downregulation of miR-7 with synthesized inhibitor inhibited cell migration in vitro, suppressed cell proliferation, and induced renal cancer cell apoptosis. Thus, miR-7 could be characterized as an oncogene in renal cell carcinoma [53].

Colorectal neoplasm is the third most common cancer and the fourth most common cancer-related cause of death worldwide, with more than 1.2 million new cases and 600,000 deaths annually [54]. MiRNAs can be used as useful biomarkers for colorectal cancer diagnosis, prognosis, and prediction of treatment response because of their several unique characteristics [55]. For example, serum miR-21, miR-29a, and miR-125b levels could discriminate early colorectal neoplasms patients from healthy controls [56]. The top 50 potential miRNAs associated with colorectal neoplasms and evidence for associations with kidney are listed in Table 4. Among the top 50 predicted candidates, 49 miRNAs were confirmed by dbDEMC. Only 1 miRNA (hsa-mir-663a) was not confirmed in the dbDEMC.

### 3.4. Prediction of Isolated Diseases and Isolated miRNAs

An isolated disease signifies a disease without any known related miRNAs or newly discovered disease. When we tested the capability of SRMDAP to predict isolated diseases, we removed all known verified miRNAs, which have been shown to be related to the predicted disease. This operation was performed to confirm that we only used the similarity information of other miRNAs-related diseases to predict candidate miRNAs associated with the given disease. Then, these candidate miRNAs were ranked according to their scores. The average AUC of SRMDAP to predict isolated disease was 0.7990. For colorectal neoplasms, we removed 143 known miRNA related to colorectal neoplasms and ranked candidate miRNAs based on the predictive result of SRMDAP. Among the top 50 predicted candidates, 49 miRNAs have been confirmed by dbDEMC. The potential candidate hsa-mir-494 is supported by the literature [PMID: 25270723]. However, hsa-mir-494 is an independent prognostic marker for colorectal neoplasm patients, and this miRNA promotes cell migration and invasion in colorectal neoplasms by directly targeting PTEN [57]. The predicted results of colorectal neoplasms are listed in Table 5.

As previously stated, an isolated miRNA is a miRNA without any known related disease, such as newly discovered miRNAs. The known verified disease-miRNA associations related to predictive miRNAs were removed to demonstrate the ability of SRMDAP to predict miRNAs without any known related disease. This procedure ensures the use of only known disease-miRNA associations and similarity information of other miRNAs to predict candidate disease. Then, these candidate diseases were ranked according to their scores. The average AUC of the SRMDAP to predict isolated miRNAs was 0.8464. The predicted results of hsa-mir-106b are listed in Table 6. For hsa-mir-106b, we removed 31 related diseases associations and ranked candidate diseases based on the predictive result of the SRMDAP. Among the top 10 predicted candidates, all diseases have been confirmed by dbDEMC, miR2Disease, or HMDD. These results demonstrate that the SRMDAP may be recommended to predict isolated diseases and miRNAs.

## 4. Discussion

The success of SRMDAP could largely be attributed to several factors. First, SRMDAP is a novel method to predict human miRNA-disease associations. This similarity measurement method does not depend on experimentally supported miRNA-disease associations to calculate the functional similarity of miRNAs and diseases. Thus, overestimation of the predictive accuracy was avoided. In SRMDAP, we proposed a density-based recommender model to integrate miRNA similarity subnetwork and disease similarity subnetwork using experimentally verified miRNA-disease associations. Second, SRMDAP incorporates miRNA-mRNA information, disease-gene information, and experimentally verified miRNA-disease associations. This characteristic improved prediction accuracy. Third, only one parameter was used to balance the contributions from miRNA similarity subnetwork and disease similarity subnetwork, and this parameter was easy to adjust. Fourth, LOOCV experiment and case studies about kidney and colorectal neoplasms demonstrated that SRMDAP had excellent predictive performance. Finally, the SRMDAP could predict isolated diseases and isolated miRNAs for disease similarity, and miRNA similarity was obtained independently on the known miRNA-disease associations.

Although SRMDAP contains several innovative concepts, this process has several limitations in its current version. First, a similarity measurement is of vital importance. Hence, miRNA similarity measurement should use more interaction information of miRNAs with other biomolecules. Disease similarity measurement should consider not only functional similarities but also semantic similarities. A fusion of more information sources can benefit the similarity measurement. Second, considering that the SRMDAP is constructed on the basis of known miRNA-disease associations, the performance of SRMDAP can be improved by obtaining more available experimentally verified miRNA-disease associations.

## 5. Conclusions

Identifying most promising miRNA-disease associations facilitates biological experimentation to save time and cost. In this work, we developed SRMDAP to predict miRNA-disease associations using established miRNA similarity subnetwork and disease similarity subnetwork based on the SimRank and density-based clustering recommender model. We integrated these similarity networks with known experimentally verified miRNA-disease associations using the density-based clustering recommender model. SRMDAP obtained average AUC of 0.8838 in LOOCV. Case studies of kidney and colorectal neoplasms were evaluated, and 49 miRNAs in the top 50 miRNAs were confirmed. SRMDAP also performed well in predicting isolated diseases and miRNAs. For colorectal neoplasms and hsa-mir-106b, all top 50 predicted miRNAs and all top 10 predicted diseases have been confirmed by dbDEMC, miRCancr, HMDD, or the literature. These results demonstrated that SRMDAP has superior performance over the other tested processes.

## Figures and Tables

**Table 1:** Global characteristic of the known miRNA-disease association network.

Number of diseases	Number of miRNAs	Number of miRNA-disease association	Avg. degree of diseases	Avg. degree of miRNAs	Max degree of diseases	Min degree of disease	Max degree of miRNAs	Min degree of miRNAs
334	475	5048	15.11	10.63	208	1	112	1

**Table 2:** Prediction result of SRMDAP and other methods for LOOCV.

Disease names	Number of related_miRNAs	AUC			
		SRMDAP	RLSMDA	KATZ	Liu's method
Carcinoma, hepatocellular	208	**0.7639**	0.6909	0.6881	0.4807
Breast neoplasms	197	**0.7776**	0.6814	0.6779	0.4147
Stomach neoplasms	174	**0.7591**	0.6635	0.6791	0.5498
Colorectal neoplasms	143	**0.7929**	0.6647	0.6895	0.4699
Melanoma	136	**0.7958**	0.6584	0.6673	0.4804
Lung neoplasms	128	**0.8874**	0.7198	0.7675	0.5243
Heart failure	120	**0.7538**	0.6608	0.6622	0.5040
Prostatic neoplasms	116	**0.8076**	0.6704	0.7054	0.5440
Ovarian neoplasms	112	**0.8732**	0.7194	0.7705	0.5382
Carcinoma, renal cell	104	**0.7367**	0.5815	0.6126	0.4932
Pancreatic neoplasms	97	**0.8687**	0.6829	0.7288	0.5355
Carcinoma, non-small-cell lung	94	**0.8322**	0.6873	0.6981	0.5470
Glioblastoma	94	**0.7686**	0.6421	0.6522	0.5644
Urinary bladder neoplasms	90	**0.7935**	0.6231	0.6635	0.5475
Carcinoma, squamous cell	78	**0.8637**	0.7179	0.7200	0.5398
Colonic neoplasms	77	**0.8271**	0.6582	0.6859	0.5490
Glioma	71	**0.8212**	0.6727	0.7146	0.5591
Esophageal neoplasms	70	**0.7789**	0.6126	0.6383	0.4781

**Table 3:** The top 50 potential kidney neoplasms-related miRNAs predicted by SRMDAP and the confirmation of these associations. Forty-nine of the top 50 kidney neoplasms-related miRNAs have been confirmed by dbDEMC. Hsa-mir-7 ranked 48th has been confirmed by the literature (PMID: 23793934).

Rank	miRNA	Evidence
(1)	hsa-mir-155	dbDEMC
(2)	hsa-mir-146a	dbDEMC
(3)	hsa-mir-17	dbDEMC
(4)	hsa-mir-125b	dbDEMC
(5)	hsa-mir-20a	dbDEMC
(6)	hsa-mir-34a	dbDEMC
(7)	hsa-mir-145	dbDEMC
(8)	hsa-mir-92a	dbDEMC
(9)	hsa-mir-16	dbDEMC
(10)	hsa-mir-126	dbDEMC
(11)	hsa-mir-18a	dbDEMC
(12)	hsa-mir-221	dbDEMC
(13)	hsa-mir-19b	dbDEMC
(14)	hsa-mir-29a	dbDEMC
(15)	hsa-mir-1	dbDEMC
(16)	hsa-mir-29b	dbDEMC
(17)	hsa-let-7a	dbDEMC
(18)	hsa-mir-19a	dbDEMC
(19)	hsa-mir-143	dbDEMC
(20)	hsa-mir-223	dbDEMC
(21)	hsa-mir-200b	dbDEMC
(22)	hsa-mir-29c	dbDEMC
(23)	hsa-mir-31	dbDEMC
(24)	hsa-let-7b	dbDEMC
(25)	hsa-mir-222	dbDEMC
(26)	hsa-mir-181a	dbDEMC
(27)	hsa-mir-210	dbDEMC
(28)	hsa-mir-199a	dbDEMC
(29)	hsa-mir-200a	dbDEMC
(30)	hsa-mir-133a	dbDEMC
(31)	hsa-mir-150	dbDEMC
(32)	hsa-mir-34c	dbDEMC
(33)	hsa-mir-146b	dbDEMC
(34)	hsa-let-7c	dbDEMC
(35)	hsa-mir-142	dbDEMC
(36)	hsa-mir-181b	dbDEMC
(37)	hsa-mir-124	dbDEMC
(38)	hsa-mir-9	dbDEMC
(39)	hsa-mir-106b	dbDEMC
(40)	hsa-let-7e	dbDEMC
(41)	hsa-mir-133b	dbDEMC
(42)	hsa-mir-196a	dbDEMC
(43)	hsa-mir-182	dbDEMC
(44)	hsa-let-7d	dbDEMC
(45)	hsa-mir-30a	dbDEMC
(46)	hsa-mir-148a	dbDEMC
(47)	hsa-mir-195	dbDEMC
(48)	hsa-mir-7	**PMID: 23793934**
(49)	hsa-mir-34b	dbDEMC
(50)	hsa-mir-24	dbDEMC

**Table 4:** The top 50 potential colorectal neoplasms-related miRNAs predicted by SRMDAP and the confirmation of these associations. Forty-nine of the 50 colorectal neoplasms-related miRNAs have been confirmed by dbDEMC. Only 1 miRNA (hsa-mir-663a is ranked 30th) is unconfirmed.

Rank	miRNA	Evidence
(1)	hsa-mir-650	dbDEMC
(2)	hsa-mir-15a	dbDEMC
(3)	hsa-mir-223	dbDEMC
(4)	hsa-mir-29b	dbDEMC
(5)	hsa-mir-518b	dbDEMC
(6)	hsa-mir-192	dbDEMC
(7)	hsa-mir-488	dbDEMC
(8)	hsa-mir-29c	dbDEMC
(9)	hsa-mir-521	dbDEMC
(10)	hsa-mir-24	dbDEMC
(11)	hsa-mir-193b	dbDEMC
(12)	hsa-mir-106b	dbDEMC
(13)	hsa-mir-15b	dbDEMC
(14)	hsa-mir-100	dbDEMC
(15)	hsa-mir-101	dbDEMC
(16)	hsa-mir-516a	dbDEMC
(17)	hsa-let-7d	dbDEMC
(18)	hsa-mir-125a	dbDEMC
(19)	hsa-let-7f	dbDEMC
(20)	hsa-let-7i	dbDEMC
(21)	hsa-mir-30c	dbDEMC
(22)	hsa-mir-214	dbDEMC
(23)	hsa-mir-513a	dbDEMC
(24)	hsa-mir-484	dbDEMC
(25)	hsa-mir-98	dbDEMC
(26)	hsa-mir-208b	dbDEMC
(27)	hsa-mir-205	dbDEMC
(28)	hsa-let-7g	dbDEMC
(29)	hsa-mir-615	dbDEMC
(30)	hsa-mir-663a	**Unconfirmed**
(31)	hsa-mir-10a	dbDEMC
(32)	hsa-mir-30b	dbDEMC
(33)	hsa-mir-20b	dbDEMC
(34)	hsa-mir-23b	dbDEMC
(35)	hsa-mir-204	dbDEMC
(36)	hsa-mir-519e	dbDEMC
(37)	hsa-mir-515	dbDEMC
(38)	hsa-mir-130b	dbDEMC
(39)	hsa-mir-296	dbDEMC
(40)	hsa-mir-134	dbDEMC
(41)	hsa-mir-132	dbDEMC
(42)	hsa-mir-520h	dbDEMC
(43)	hsa-mir-128	dbDEMC
(44)	hsa-mir-572	dbDEMC
(45)	hsa-mir-30d	dbDEMC
(46)	hsa-mir-197	dbDEMC
(47)	hsa-mir-151a	dbDEMC
(48)	hsa-mir-654	dbDEMC
(49)	hsa-mir-138	dbDEMC
(50)	hsa-mir-495	dbDEMC

**Table 5:** The top 50 potential isolated diseases predicted of colorectal neoplasms. Forty-nine of the top 50 colorectal neoplasms-related miRNAs have been confirmed by dbDEMC. miRNA hsa-mir-494, which is ranked 45th, has been confirmed by literature.

Rank	miRNA	Evidence
(1)	hsa-mir-29b	dbDEMC
(2)	hsa-mir-15a	dbDEMC
(3)	hsa-mir-223	dbDEMC
(4)	hsa-mir-29c	dbDEMC
(5)	hsa-mir-106b	dbDEMC
(6)	hsa-let-7d	dbDEMC
(7)	hsa-mir-24	dbDEMC
(8)	hsa-mir-100	dbDEMC
(9)	hsa-mir-214	dbDEMC
(10)	hsa-let-7f	dbDEMC
(11)	hsa-let-7g	dbDEMC
(12)	hsa-let-7i	dbDEMC
(13)	hsa-mir-15b	dbDEMC
(14)	hsa-mir-125a	dbDEMC
(15)	hsa-mir-205	dbDEMC
(16)	hsa-mir-101	dbDEMC
(17)	hsa-mir-30b	dbDEMC
(18)	hsa-mir-30c	dbDEMC
(19)	hsa-mir-192	dbDEMC
(20)	hsa-mir-23b	dbDEMC
(21)	hsa-mir-20b	dbDEMC
(22)	hsa-mir-132	dbDEMC
(23)	hsa-mir-138	dbDEMC
(24)	hsa-mir-193b	dbDEMC
(25)	hsa-mir-302b	dbDEMC
(26)	hsa-mir-296	dbDEMC
(27)	hsa-mir-151a	dbDEMC
(28)	hsa-mir-204	dbDEMC
(29)	hsa-mir-196b	dbDEMC
(30)	hsa-mir-10a	dbDEMC
(31)	hsa-mir-30d	dbDEMC
(32)	hsa-mir-212	dbDEMC
(33)	hsa-mir-128	dbDEMC
(34)	hsa-mir-302a	dbDEMC
(35)	hsa-mir-191	dbDEMC
(36)	hsa-mir-302c	dbDEMC
(37)	hsa-mir-197	dbDEMC
(38)	hsa-mir-629	dbDEMC
(39)	hsa-mir-99b	dbDEMC
(40)	hsa-mir-181c	dbDEMC
(41)	hsa-mir-130b	dbDEMC
(42)	hsa-mir-30e	dbDEMC
(43)	hsa-mir-181d	dbDEMC
(44)	hsa-mir-98	dbDEMC
(45)	hsa-mir-494	**PMID:** **25270723**
(46)	hsa-mir-452	dbDEMC
(47)	hsa-mir-365a	dbDEMC
(48)	hsa-mir-32	dbDEMC
(49)	hsa-mir-184	dbDEMC
(50)	hsa-mir-424	dbDEMC

**Table 6:** The top 10 potential isolated miRNA predicted of hsa-mir-106b. All of the top 10 hsa-mir-106b related diseases have been confirmed by dbDEMC, miR2Disease, or HMDD databases.

Rank	Disease	Evidence
(1)	Carcinoma, hepatocellular	HMDD
(2)	Breast neoplasms	dbDEMC, miR2Disease, HMDD
(3)	Stomach neoplasms	HMDD
(4)	Colorectal neoplasms	dbDEMC, miR2Disease
(5)	Lung neoplasms	dbDEMC, miR2Disease
(6)	Melanoma	dbDEMC, HMDD
(7)	Ovarian neoplasms	dbDEMC, HMDD
(8)	Prostatic neoplasms	HMDD
(9)	Heart failure	miR2Disease, HMDD
(10)	Pancreatic neoplasms	dbDEMC, miR2Disease
